# Could care giving have altered the evolution of human immune strategies?

**DOI:** 10.1093/emph/eoae004

**Published:** 2024-01-25

**Authors:** Bethany L P Gilbert, Sharon E Kessler

**Affiliations:** Department of Psychology, Faculty of Natural Sciences, University of Stirling, Stirling FK9 4LA, UK; Department of Psychology, Faculty of Natural Sciences, University of Stirling, Stirling FK9 4LA, UK

**Keywords:** immune strategy, human evolution, care giving, healthcare, life history theory, agent-based model

## Abstract

Life history theory indicates that individuals/species with a slow pace of life invest more in acquired than innate immunity. Factors that decrease the pace of life and predict greater investment in acquired immunity include increased nutritional resources, increased pathogen exposure and decreased risk of extrinsic mortality. Common care behaviors given to sick individuals produce exactly these effects: provisioning increases nutritional resources; hygiene assistance increases disease exposure of carers; and protection can reduce the risk of extrinsic mortality to sick individuals. This study, therefore, investigated under what conditions care giving behaviors might impact immune strategy and pace of life. The study employed an agent-based model approach that simulated populations with varying levels of care giving, disease mortality, disease transmissibility, and extrinsic mortality, enabling measurements of how the immune strategy and age structure of the populations changed over evolutionary time. We used multiple regressions to examine the effects of these variables on immune strategy and the age structure of the population. The findings supported our predictions that care was selected for an acquired immunity. However, the pace of life did not slow as expected. Instead, the population shifted to a faster, but also more cost-intensive reproductive strategy in which care improved child survival by subsidizing the development of acquired immune responses.

## INTRODUCTION

The timing of the origins of care for sick and injured individuals during human evolution is not definitively known and is controversial [1–5]. However, when we look across species, there are strong continuities between human behaviors and patterns observed in other species, suggesting that the origins of care are far older than our lineage.

Across the animal kingdom, occasional care giving for sick and injured individuals is widespread [3, 6]. For many species, this involves behaviors like social grooming (e.g. primates, ungulates, birds, insects), remaining close to individuals who are unable to move quickly with the group (e.g. primates, elephants, giraffe, pinnepeds, mongoose), or provisioning individuals who are unable to hunt (mongoose, lions, foxes, giant otters) [3]. Some species exhibit striking behavior patterns that are specific to the environments that they inhabit or create, for example, aquatic mammals that have been documented lifting conspecifics to the surface to breathe [7, 8] or nest sanitation behaviors that may reduce pathogen transmission in birds and eusocial insects [3]. Within species, the specific care behaviors that are given to sick/disabled individuals frequently overlap with behaviors given to vulnerable dependent young, leading various researchers to suggest that care for sick and injured individuals may be an extension or co-optation of offspring care [3, 6, 9–11]. Across species, the characteristics that predict care giving appear to be complex interactions between environmental and social factors, with more frequent care occurring in species that engage in high levels of niche construction (e.g. nest building) or cooperative breeding (e.g. allocare and allofeeding) [6].

Given that humans engage in more complex niche construction and breed more cooperatively than our closest living relatives, the extant apes, it seems likely that ancestral hominins were capable of at least as much care as extant apes and that, since diverging from other apes, the amount of care we give and its complexity has increased greatly [6, 12–18]. Fossil records provide evidence of individuals surviving injuries, illness and disabilities [1, 2, 5, 19, 20]. Although the individuals might have been able to survive without care, it is difficult to rule out the possibility that substantial, long-term care may have been given [4, 5, 20–31]. The genus *Homo* has been suggested to have possessed a suite of social and cognitive abilities like tendencies to be prosocial and cooperative, which may have made care increasingly widespread [4, 5, 32, 33]. However, regardless of whether the fossil record provides evidence of increasing care giving in our lineage, it is likely that whenever this occurred, it would have had two important effects.

First, care would have generated substantial immune costs for carers [3]. Many (but not all) forms of care require the care-giver to be exposed to the socially transmittable pathogens from which the recipient may be suffering [3]. Thus, as care evolved, it would have exerted pressures on the immune system to withstand the exposures experienced by care-givers [34]. Second, care would have potentially shielded sick individuals from some of the selective pressures exerted by pathogens either directly or indirectly, for example, poor nutrition due to reduced foraging abilities, increased predation risk due to reduced mobility, and so on.

McDade *et al.* [35] provide a theoretical framework that can be applied to care giving and used to predict how these two effects of care giving relate to life history trade-offs, and, as a result, how they would also impact the evolution of immune strategies in our lineage. A key assumption in life history theory is the ‘allocation rule’, which states that energy is limited and individuals must make trade-offs between life functions [36]. As a result, there are two main strategies for maximizing reproductive success [36–38]—a fast pace of life (short life-span, short development, short inter-birth intervals and little/no parental investment) and a slow pace of life (long life-span, long development, long inter-birth intervals and substantial parental investment) [39, 40]. There are different energetic costs to the development, initiation and deployment of different elements of the immune system [35,41,42] (see Table 1 for summary). Therefore, these two pace-of-life strategies are likely to correspond to differences in immune strategy [35].

**Table 1.** Summarized Costs of Immunity-Source adapted from McDade *et al.* [35] and Klasing *et al.* [42]

	Costs		Effectiveness		
	Developmental	Activation	Collateral	Novel exposure	Secondary exposure
Innate immunity	Low	High	Medium	Good	Good
Acquired immunity	Very high	Low	Low	Poor	Excellent

McDade *et al.* [35] focus on the subdivision and trade-offs between investing in acquired and innate immune responses. Acquired immunity primarily employs T and B lymphocytes, launching a highly specific immune response and develops an immunological memory from which future responses can be launched. Innate immunity uses a broader range of cells, launching more a general immune response [43, 44].

The development of the acquired immune system is particularly costly because of the B and T lymphocyte screening process in which 95% of the lymphocytes are deconstructed [45]. This inefficient system requires a considerable amount of energy and resources [45–47]. Like acquired immunity, the development of innate immunity also requires chemical substrates. However, the development of innate immunity is more efficient and occurs gradually [35, 42, 47]. Very often, innate and acquired immune responses occur simultaneously and it can be difficult to ascertain the energetic costs of each individual response [42]. However, the deployment of an acquired immune response, particularly in response to reinfection, uses fewer chemical substrates and is, therefore, overall less costly [35, 42]. The deployment of an innate immune response is likely to be much more costly as it uses a greater repertoire of cells that initiates energetically costly processes such as the acute phase and fever [35, 48].

McDade *et al.* [35] proposed a framework encompassing the main factors impacting life history strategy and made predictions for how they would also impact immune strategy. They hypothesized that (i) decreased extrinsic mortality, (ii) increased pathogen exposure and (iii) increased nutritional resources were all environmental factors that would promote a slow pace of life and increase investment in acquired immunity relative to innate immunity. Extrinsic mortality is the likelihood of death caused by exogenous sources in the environment and is impacted by factors such as predation [49–51]. In environments where extrinsic mortality is high, individuals should be selected to invest in innate immune responses because they may not live long enough to use the key advantage of acquired immunity: a specific immune response [40]. Pathogen exposure is the number and variation of pathogens to which an individual is exposed. The acquired immune system is often referred to as being exposure driven and, therefore, increased pathogen exposure is predicted to increase investment in acquired immunity [34, 35]. Nutritional resources are the number of calories an individual consumes and the extent to which the diet consists of the necessary nutritional requirements. Nutritional resources allow individuals to develop the more costly acquired immune responses by providing the necessary chemical substrates and energy to support its somewhat inefficient development. Increasing nutritional resources is, therefore, expected to increase investment in acquired immunity [52, 53].

The care giving literature demonstrates that many forms of care impact extrinsic mortality, pathogen exposure and nutritional resources [3, 54–56], thus under the McDade *et al.* [35] framework, care giving would be expected to influence trade-offs between investment in acquired and innate immunity. Specifically, care giving may reduce the extrinsic mortality risk (e.g. protection reducing predation on sick individuals), increase pathogen exposure (when carers are exposed), and increase nutritional resources available to sick/injured individuals via provisioning [3, 54–56]. Through these effects, increasing levels of care giving in the human lineage should have selected for a slower pace of life and a shift toward greater investment in acquired immunity.

We investigate these questions using a creative, agent-based modeling approach. Agent-based modeling is a flexible, quantitative approach that can be used to investigate evolutionary processes in simulated environments. It is a powerful tool for developing and testing novel theories because it allows researchers to conduct controlled comparisons that would be impossible in the real world. Here, we examined how care giving interacted with disease characteristics and levels of extrinsic mortality to select for shifts in the pace of life and immune strategy of the population. We coded a population of hominins to move, forage, provide care, invest in acquired or innate immunity, reproduce and die. We systematically varied disease characteristics, extrinsic mortality and the level of care that hominins provided, enabling us to disentangle the effects of each with a precision that would not be possible with populations evolving in real time.

## METHODS

The full model code and model description are included in Appendices A and B, respectively. All coding was conducted in Netlogo (version 6.2.0). agent-based modeling aims to simulate simplified versions of life to assess relationships between variables in controlled environments. An agent is a being that can follow a set of instructions provided by the code. A patch is grid cell that can follow a set of instructions and act as the ‘ground’ traversed by the agents. A time-step is the time it takes agents and patches to carry out all procedures which, in the case of this model, represents one year. A model run is the number of time-steps the agents and patches are asked to perform.

### Ethical considerations

The study was carried out under the guidelines of the British Psychology Association and approved by the University of Stirling General University Ethics Panel (# EC 2021 2271 1989, Date: 19 May 2021). As all the data were generated from computer simulations, no live participants were used.

### Model overview

Full details of the components of the model are described below. The basic concepts of the model are as follows. Its overarching aim was to assess whether prioritizing acquired immunity was more advantageous than prioritizing innate immunity in circumstances of varying benefits of care and varying conditions of disease severity and extrinsic-mortality because these were the factors that would impact an individual’s pace of life. A population of agents was coded to move around in ways that represented hominins in a fission-fusion dynamic (described in more detail below), on a grid of patches that represented their habitat [55, 57]. One time-step represented 1 year and each model ran for 750 time-steps, representing approximately 50 generations. Demographic data such as the number of hominins, the number of times hominins reproduced and disease prevalence at each time step were also recorded.

Hominins collected resources from the grid cells/patches and used their resources as a form of energy currency. A disease was introduced to the hominins who could then invest their resources in overcoming the disease and reproducing asexually. The number of resources care-givers provided to an infected relative varied between model runs and is referred to as *care intensity*. The hominins were pre-coded to prioritize the use of either innate or acquired immunity when combatting the disease. This is referred to as their *immune strategy*. The immune strategy was coded to be heritable thereby allowing an assessment of which immune strategy was most advantageous over the course of many generations in the model. Over many generations, hominins with the most successful immune strategy (prioritizing investment in innate or acquired immunity) were expected to reproduce more, causing that strategy to become more prevalent.

### Model details

#### Spatial lay out

The world grid was set at 40 × 40 grid cells, each of which represents 5 km², making the habitat 8000 km². Carrying capacity was set to 200 hominins. This was based on a paper by Layton *et al.* [58], which collated data regarding the distance materials were moved from their source during various periods of hominin evolution, ethnographic data on hunter-gatherer daily foraging ranges, population densities collated by the authors and fossil hominin morphology [58]. The paper reported predicted figures for European Neanderthals during the middle and upper paleolithic period compared to ethnographic data on modern Arctic hunter-gatherers. Layton *et al.* [58] proposed that the maximum distance for a community of 145 individuals (as predicted by Aiello and Dunbar [59]) would result in a community area of a maximum of 29 000 km² but would average at 10 150 km². Community sizes greater than 150 are unlikely to have been exhibited in ancestral hominins but are included to investigate fully the demographic impact of care giving [57, 59, 60].

#### Grid cells (patches)

Patches provided resources to the hominins (see resource score explanation below). The resources available from a patch represented ancestral hominin foraging opportunities with large game and other micronutrients in the form of fruit and nuts [61]. A hominin could only move to a patch within a radius of five patches that had a sufficient number of resources. If the patch had 10, it was available to both adult and child hominins. If a patch had between 5 and 10 resources, it would be available to child hominins only. When an adult hominin (over 15 years old) moved to a patch, their resource score increased by 10. The patch’s variable ‘resources’ decreased to zero and the patch became unavailable to all other hominins. When a child hominin (under 15 years old) moved to an ‘available’ patch, they only consumed five resource points, the patch’s resources decreased by five. When the number of recourses on a patch was less than 10, it reset to 10. This only occurred once a time step, prior to foraging meaning that if a patch had been ‘foraged’, it was unavailable for the rest of the time step. Therefore, this cycle represented a yearly fruiting/animal reproduction cycle with a population under the carrying capacity of the environment. If the number of hominins in the model was greater than 150, patches stopped resetting their resources to 10, representing the population reaching the carrying capacity of the environment.

#### Hominins

Hominins were coded to forage, age, reproduce and provide care to infected relatives. Hominins had a resource score variable and an age variable. The resource score acted as an energy currency and could be used to overcome infection, reproduce and provide care. Hominins did not start reproducing until adulthood at age 15, stopped reproducing at 45 and died at 60 (unless killed by disease-mortality, extrinsic-mortality or starvation earlier), which was based on demographic findings regarding modern hunter gatherer communities [62]. A threshold of 20 resources was placed on reproduction to represent the unlikelihood of malnourished hominins reproducing successfully. Hominins reproduced asexually and offspring had a 75% chance of inheriting their parent’s immune strategy to introduce variation. Offspring were identical to their parents in all other aspects. If a hominin was coded to prioritize investment in acquired immunity then, provided they had the available resources to combat their infection, there was a 75% chance they would use acquired and a 25% chance they would use innate immunity. If the hominin’s investment strategy prioritized innate immunity, the opposite occurred. We did not code hominins to use or inherit only acquired or innate immune strategies to reflect that the two elements of the immune system do not work in isolation and because of this both need to be maintained in the model over evolutionary time. While trade-offs between acquired and innate immunity are also predicted to occur during development, with the plasticity of developmental processes responding to environmental conditions, we consider developmental plasticity to be beyond the scope of the model and do not model it explicitly.

#### Resource score

This was the number of resources a hominin had collected. Ten resource points represented the number of calories consumed per year in conditions where food was readily available. According to the findings of Hill and Hurtado [62] the number of calories consumed by adult men in hunter gatherer societies is between 2000 and 3000 calories/day and for women, between 1000 and 2000 calories/day. As the hominins in the model were asexual, they consumed 2000 calories a day. Hominins collected 10 resource points per time step which equaled 730 000 calories (365 × 2000). Hominins with enough resource points could overcome infection, provide care and reproduce. The cost of reproduction was set at 20 resource points. Therefore, the cost of reproduction was 2 years’ worth of resources. Hill and Hurtado [62] found that the average inter-birth interval for the ache hunter gatherer community was 37 months [62]. However, it is reasonable to suggest that the inter-birth interval would have been shorter in ancestral hominins [63]. Therefore, the inter-birth interval for hominins in the model was shortened to 24 months. The cost of investing in innate immunity was 5 resource points. The cost of investing in acquired immunity was 10 resource points to represent the greater activation cost of acquired immunity [35].

The cost of initiating an immune response can be high as literature suggests that individuals in hunter-gatherer communities experience infections that may prevent them from foraging for over a month [61, 64]. Although immune responses generally include both innate and acquired responses, in our model hominins use one or the other, in order to disentangle the strategies over evolutionary time.

#### Immune strategy and combatting disease

When a hominin becomes infected, the model generates a random number between 0 and 100. If the hominin prioritizes innate immunity as their immune strategy and the randomly generated number is between 0 and 75, the hominin will use innate immunity to combat infection. Therefore, if a hominin is using the innate immune strategy, there is a 75% chance that they will use innate immunity to combat infection and a 25% chance that they will use acquired immunity to combat infection. As hominins were coded to prioritize innate or acquired immunity, this decision-making process was mirrored for prioritizing acquired immunity. Therefore, the model provides an opportunity for individuals to use the strategy that they do not prioritize.

#### Combatting infection using innate immunity

First, the hominin completed the disease mortality section of the procedure (described below in the disease mortality section). Hominins who used innate immunity to combat infection used five resource points to overcome the infection. If the hominin did not have five resource points, they remained infected until they had the five resource points to combat infection. The hominins did not remain immune to infection. The first deployment of an innate immune response required less resources than the first deployment of acquired immunity (described below) thereby simulating the relatively low cost of developing a nonspecific immune response, such as a fever. However, any subsequent deployments of innate immunity also cost five resource points each time, representing the relatively larger cost of repeated deployments relative to repeated deployments of acquired immunity.

#### Combatting infection using acquired immunity

First, the hominin completed the disease mortality section of the procedure (described below in the ‘Disease mortality’ section). When a hominin used acquired immunity to combat infection they first checked if they were infected and if they recorded the infection. If the hominin had previously been infected and recorded it, they overcame the infection, costing them one resource point. If the hominin did not record the infection, they remained infected until they had more than 10 resource points, at which point they used 10 resource points to overcome the infection and record it. The deployment of acquired immunity required the use of more resource points thereby simulating the greater upfront cost of mounting the initial specific immune response. However, once acquired immunity had been employed the hominin remembered the infection for five time-steps, representing 5 years of immunity. Maintaining the immunity gained by combatting the disease with acquired immunity costs 1 resource point per-time step. The memory for a disease that is developed when an acquired immune response is employed is usually long-term [65]. However, for the purposes of the model, the disease is forgotten after the five-time steps, simulating waning immunity in which the individual is susceptible to similar infections over time.

### Disease

Disease-mortality was the chance of the disease killing hominins and was set at 0%, 5%, 10% and 15%. Thus, disease mortality varied to approximate the disease mortality rates in a range of common, persistent diseases. For example, case fatality rates in a disease such as COVID-19 were around 17.62% among hospitalized patients at the height of the pandemic [66]. As mentioned earlier, the hominin performed this procedure once they had chosen which immune strategy they were going to use. The model then generated a random number between 0 and 100 for each hominin. If the random number was less than the pre-set disease mortality, the hominin died. The hominins did not have the chance to combat infection prior to the disease mortality procedure. Therefore, hominins who remained infected because they did not have the necessary resources to overcome infection, experienced a much greater chance of dying in the next time step. Transmissibility was the percentage chance of the hominin catching the infection from another hominin within the infection radius (five patches) and was set at 25%, 50% or 75% in order to sample the range of possible values. Transmissibility was not set to 0 or 100 because the model focuses specifically on socially transmitted diseases and 0% or 100% transmission rates are unlikely.

The infection radius encompassed the patches immediately surrounding the patch occupied by an infected hominin. It was set to five grid cells, with each grid cell representing 5 km^2^, because hominins can travel up to 25 km in a day and are, therefore, likely to have interacted within that range on a given day within the course of the year [58]. Consequently, hominins could become infected if there was an infected hominin within that radius. The model generated a random number in the form of a percentage for each hominin in the infection radius of 25 km. If the random number was less than the pre-set disease transmissibility, the non-infected hominin became infected, regardless of resource points.

It is worth noting that while the model’s time steps represent a year, infections are calculated using the spatial scale of a day because they use an infection radius of a hominin’s day range. In this respect, the probabilities of transmission represent a year’s worth of transmission risk (because the agents do not do 365 days per time-step), but when they are infectious, they only transmit over 1/365th of the area they might possibly travel through. We aimed to capture the yearly risk, which was important to create a disease that could be sustained over an evolutionary time-scale, yet retain some realism in that individual infected agents are not likely to transmit over the entire geographic area that they traverse in the year (up to 9125 km, if they travel 25 km for 365 days per year). Thus, transmissibility represents the probability of transmission on ***any*** given day of the year-long time step.

### Extrinsic mortality

Extrinsic mortality was the percentage chance of hominins who had not received care dying and was set at 0%, 1%, 5% or 10%. In this way, extrinsic mortality simulated the impact of harsh conditions removing uncared for individuals. If the number of hominins in the model was greater than 150, the model generated a random number for each hominin. If the number was less than the pre-set extrinsic-mortality and the hominin had not received care, the hominin died. Extrinsic mortality factors such as predation are expected to increase with high population densities that approach the carrying capacity of the environment [67]. Therefore, extrinsic mortality only occurred when the number of hominins in the model was above 150. Paleoanthropological reconstructions suggest that community sizes exceeding 150 would have been large, and, within the model, those communities were approaching the limits set by the carrying capacity of the environment (200) [57, 59, 60, 68].

### Care intensity

Hominins cared for infected relatives by providing them with resources and protection from extrinsic-mortality, representing provisioning and protection. The care intensity varied the number of resource points care-givers gave to infected relatives. The care intensity was set to 0, 10 or 20 resources. As 10 resource points are 1 year’s worth of resources, 20 represent up to 2 years’ worth of resources, representing extremely extensive provisioning. On receiving resources from the care giver, an infected hominin used them to assist the mounting of either an acquired or innate immune response. Consequently, hominins who received care were more likely to recover. If a hominin received care, they became safe from extrinsic mortality, representing protection as well as provisioning. This protection only lasted for the duration of the time step. Furthermore, if the care intensity is set to 0, there would be no transfer of resources, but the recipient would still be safe from extrinsic mortality. Evolutionary theory dictates that individuals care for their genetic relatives as a method of increasing the chances of shared genetics being passed on to offspring [6, 69–71]. Hominins in the model provide care via the parent–offspring bond because previous literature shows that mother–offspring relationships are commonly recognized, particularly in mammals, and aid frequently goes to maternal kin [3]. Therefore, if care is only provided by the parent to an offspring or vice versa, the recipient is always kin for the purposes of the model [24]. Provisioning was selected as a major element of care because it is expected to have been important in hominins and has been recorded in nonhuman animals [64]. For example, tolerance of food theft/begging for sick or ill individuals has been documented in giant otters (*Pteronura brasiliensis*) and food sharing for sick and injured family members has also been documented in wild mongooses (*Herpestes parvula*) [54, 56].

### Initialization

Prior to the commencement of each model run, 100 hominins were placed randomly in the landscape to represent a medium size hominin community. Each hominin was randomly assigned an investment strategy and an age between 0 and 60. For five time steps, all hominins were allowed simply to collect resources. After five-time steps, the disease selected 50% of hominins at random and infected them. If the disease became extinct another 50% of hominins were selected at random and infected. The aim of the model was to explore the immune responses of the population, not to examine whether diseases successfully become established. Therefore, in order to ensure that the disease would become established, we seeded it into the population at a very high rate and if the disease became extinct, it was immediately re-introduced. As each time-step represents a year, this is similar to how large percentages of populations can be infected or exposed to a circulating respiratory virus (e.g. covid-19) within a year or less [72].

### Model run

After initialization the hominins age and forage. All infected hominins then transmitted the disease to non-infected hominins in a radius of five grid cells (25 km^2^) according to the transmissibility probability. The hominins then provided care to their infected relatives. If the number of hominins in the model was greater than 150, the extrinsic mortality procedure occurred. All infected individuals then performed the combatting disease procedure that included the disease mortality procedure. If hominins survived the disease mortality section of the procedure, they then combatted their infection using either innate or acquired immunity. Hominins that survived (having deployed their immune response) and still had more than 20 resource points then reproduced as long as the carrying capacity in the model had not been reached. This process occurred for each of the 750 times steps in each model run.

**Figure 1.**
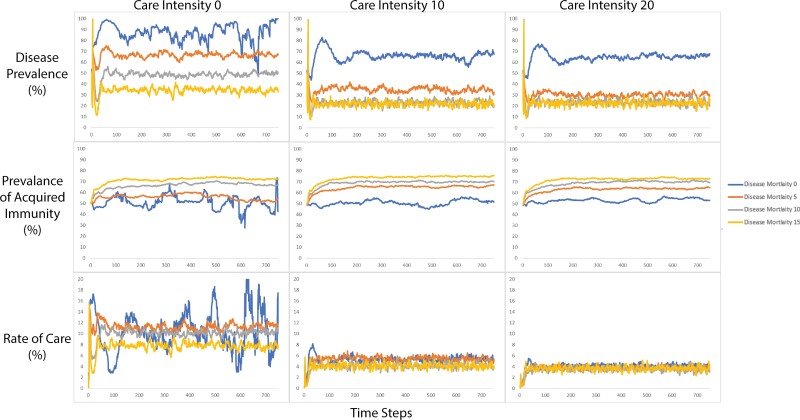
The top row shows a change in disease prevalence over time as produced by disease mortality varying in severity from 0 to 15 (colors), under the 3 care intensities (columns). The middle row shows a change in the prevalence of acquired immunity as produced by disease mortality in varying severity from 0 to 15 (colors), under the 3 care intensities (columns). The bottom row shows a change in the rate of care over time as produced by disease mortality varying in severity from 0 to 15 (colors), under the 3 care intensities (columns)

**Figure 2.**
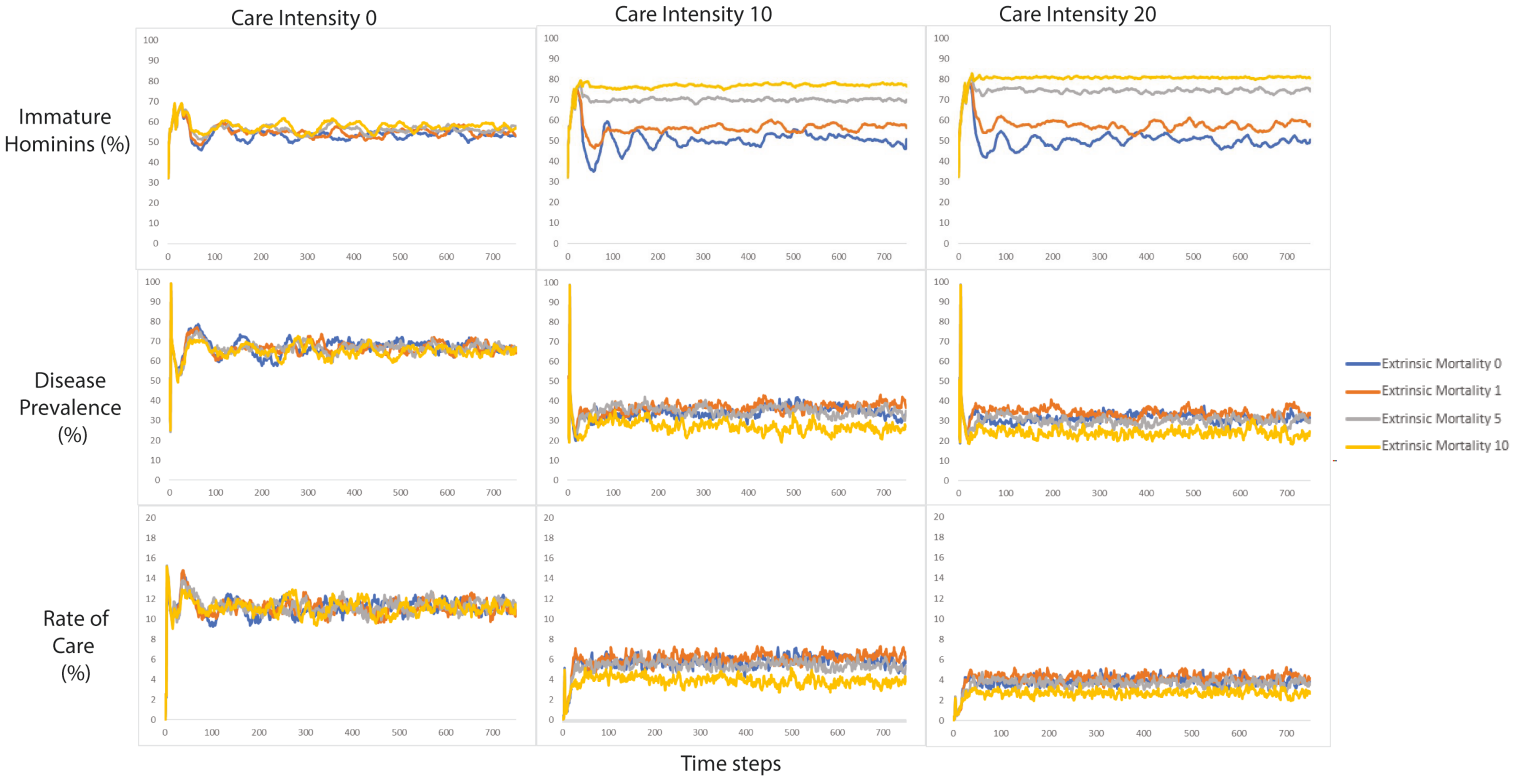
The top row shows chance in the percent of immature hominins over time as produced by extrinsic mortality in varying severity (0, 1, 5, 10), under the three care intensities (columns). The middle row shows a change in the disease prevalence as produced by extrinsic mortality in varying severity (0, 1, 5, 10), under the three care intensities (columns). The final row shows a change in the rate of care as produced by extrinsic mortality in varying severity (0, 1, 5, 10), under the three care intensities (columns)

**Figure 3.**
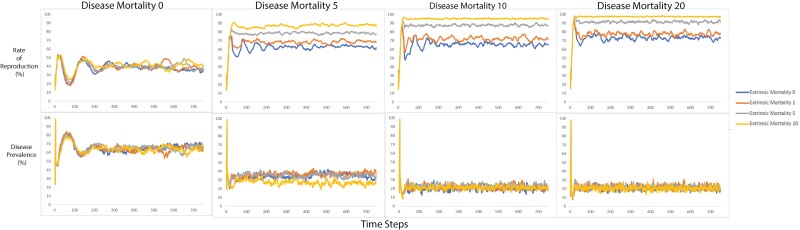
The top row shows a change in the rate of reproduction over time as produced by extrinsic mortality in varying severity (0, 1, 5, 10), under the four disease mortalities (columns). The final row shows a change in disease prevalence as produced by extrinsic mortality in varying severity (0, 1, 5, 10), under the four disease mortalities (columns)

At each time step, the following output variables were recorded: the number of hominins, the number of hominins that reproduced, the number of infected hominins, the number of hominins aged 0–14 (immature hominins) and 15–60 (mature hominins), the percentage of hominins who prioritized innate immunity and the percentage of hominins who prioritized acquired immunity. Outputting these variables at each time step and at the end of the run enabled us to compare how the populations differed after 750 time-steps under varying conditions of care intensity, disease mortality, transmissibility and extrinsic mortality. Values of care intensity, disease mortality, transmissibility and extrinsic mortality were fixed and did not change over the course of a run. All possible variable combinations were run 100 times. Care intensity varied from 0 to 20 in intervals of 10. Disease mortality varied between 0 and 15 in intervals of 5. Transmissibility varied from 25 to 75 in intervals of 25. Extrinsic mortality was set to 0, 1, 5 or 10. This produced 144 possible variable combinations. As each variable combination was repeated 100 times, we had a total of 14 400 runs. The averages were calculated across runs with identical variable settings so that the 100 time-steps 1 were averaged together to produce one average time step 1, the 100 time-steps 2 were averaged together to produce one average time-step 2, and so on, until we had produced on one complete average run for every possible combination of variables. These average runs were produced in order to present a visualization of the patterns in a highly stochastic dataset over time (Figs. 1–3). As the aim of the model was to assess the evolution of the hominin population, we averaged the final 100 time steps of each variable combination. Consequently, the statistical analysis was conducted on the average of the last 100 time steps of each outcome variable. Each row of data included the disease and extrinsic mortality parameters set for the model run and the average of the final 100 runs of the outcome variable. Runs where the population died out prior the model did not complete 750 runs because either the population died out or disease prevalence reached 100% and the model stopped where excluded from analysis. We excluded runs where the population died out prior the model did not complete 750 runs because either the population died out or disease prevalence reached 100% and the model stopped (see Table 2).

**Table 2.** The model produced a total of four runs in which the population either did not survive 750 time steps or disease prevalence reached 100% and the model stopped

Disease mortality	Transmissibility	Extrinsic mortality	Care intensity
0	25	5	0
0	50	0	0
0	75	0	0
0	75	5	0

## ANALYSIS

We ran a series of multiple regressions using the following variables derived from the model parameters: *disease mortality* (set at 0%, 5%, 10% or 15% chance of death), *transmissibility* (set at 25%, 50% or 75% chance of infection by another hominin within the infection radius), *extrinsic mortality* (set at 0%, 1%, 5% or 10%chance of death) and *care-intensity* (set at 0, 10 or 20 resources transferred during care). In addition, we calculated several other emergent variables from the model data. *Disease prevalence* was the percentage of the population that was infected in a time step. *Rate of care* was the percentage of infected individuals who received care during a time step. *Innate immunity* and *acquired immunity* were the percentages of hominins prioritizing (not necessarily using) innate and acquired immunity in that time step, respectively. *Percent immature hominins* and *percent mature hominins* were the percentage of hominins younger than 15 and 15 or older in the population in a time step, respectively. *Rate of reproduction* was the percentage of mature hominins who reproduced during a time step. As the aim of the study was to assess the end result of the populations’ evolution, we averaged the final 100 time-steps representing approximately the last 7 generations to be used on analysis.

We assessed three main areas of interest: (1) the interaction of disease characteristics, extrinsic mortality and care on disease prevalence; (2) how disease, extrinsic mortality and care influence immune strategy; and (3) how disease, extrinsic mortality and care influence pace of life. We did this in the manner set out below:

(1) We used a multiple regression to test the effects of disease mortality, transmissibility, care intensity and extrinsic mortality on disease prevalence.
(2) We expected care intensity to have nonlinear effects based on if, and how much, provisioning occurred (resource transfer during care intensity 0, 10 and 20). Therefore, we conducted two separate multiple regressions at each care intensity and tested the effects of transmissibility, disease mortality, disease prevalence, extrinsic mortality and rate of care on acquired immunity and on innate immunity.
(3) To investigate changes in the pace of life, we ran three multiple regressions. The first two tested the effects of transmissibility, disease mortality, disease prevalence, extrinsic mortality, care intensity and rate of care on the percentage of immature and mature hominins. The third tested the effects of transmissibility, disease mortality, disease prevalence, extrinsic mortality care intensity and rate of care on the reproductive rate.

All analysis was conducted in R Studio, (version 4.3.0), using the tidyverse, lm.beta and readxl packages. The alpha was 0.05. Graphs were produced using excel from Microsoft Office 365.

## RESULTS

Of the 144 average runs, 140 (97%) ran to completion (Table 2). Figs. 1–3 show the effects of the different parameter sets on the emergent variables generated by the model over time.

### Interaction of disease characteristics, extrinsic mortality and care on disease prevalence

A multiple regression was used to assess the impact of disease mortality, transmissibility, care intensity, extrinsic mortality and rate of care on disease prevalence (*M* = 42.1%, SD = 21.7%) during the last 100 time steps. The regression showed that the predictors accounted for 80% of the variance *F*(5,134) = 106.4, *P* = 2.2 × 10^−16^ (see Table 3). Rate of care was positively associated with disease prevalence. This is likely because when disease prevalence is high, more hominins are in need of care, producing a higher rate of care.

**Table 3.** The effects of disease mortality, transmissibility, care intensity, extrinsic mortality and rate of care on disease prevalence

Predictor	Beta	*T*	*P* value
Transmissibility	0.04	−0.77	0.45
Disease mortality	0.15	−19.2	**2.2 × 10** ^**−16**^
Extrinsic mortality	0.21	−1.21	0.23
Rate of care	0.52	3.02	**3.1 × 10** ^**−4**^
Care intensity	0.18	−4.42	**2.04 × 10** ^**−5**^

Bold text highlights significant *P*-value (*P* < 0.05).

Disease mortality was negatively associated with disease prevalence. This is likely to have occurred because as mortality increased, fewer hominins survived the infection to transmit it. Care intensity appears to have served to control the disease—as care intensity increased (thus increasing transfer of resources to the infected individual), the individual used those resources to overcome the infection and consequently did not transmit it during the following time step.

Surprisingly, transmissibility itself did not significantly predict disease prevalence. Transmissibility was the percentage chance of the disease infecting the individual within a 5-patch radius. However, because the disease was re-seeded into the population if it died out, by re-infecting 50% of the population, this process was likely more impactful than the differences in the transmissibility parameter, rendering transmissibility unimportant in the model.

Extrinsic mortality was not a driver of prevalence as it occurs at both high and low prevalence, affecting both infected and healthy hominins. While those who have received care are protected from extrinsic mortality, the effects of this are likely accounted for within the role that care intensity played in reducing prevalence (see Fig. 1).

### Selection on immune strategy

#### Care intensity 0

A multiple regression was used to assess the impact of disease mortality, transmissibility, extrinsic mortality, disease prevalence and rate of care on acquired immunity (*M* = 64.5%, SD = 8.71%) when care intensity was set to 0. The regression showed that the predictors accounted for 54% of the variance *F*(5, 38) = 9.23, *P* = 8.17 × 10^−6^ (see Table 4). While the overall regression was significant, none of the predictors had a significant impact on the prevalence of acquired immunity when care intensity was set to 0.

**Table 4.** The effects of disease mortality, transmissibility, disease prevalence, extrinsic mortality and rate of care on the prevalence of hominins who prioritized acquired immunity when care intensity was set to 0

Predictor	Beta	*T*	*P* value
Transmissibility	0.05	−0.11	0.91
Disease mortality	1.15	1.76	0.09
Extrinsic mortality	0.03	−0.83	0.41
Disease prevalence	0.59	−1.07	0.29
Care intensity	0.30	0.87	0.39

As hominins prioritized either acquired or innate immunity, the impact of the predictors on innate immunity mirrors that of acquired immunity *F*(5, 38) = 9.23, *P* = 8.17 × 10^−6^ (see Table 5). Thus, in the model, the effects of the predictors on innate immunity (*M* = 35.5%, SD = 8.71%) were the opposite of those on acquired immunity.

**Table 5.** The effects of disease mortality, transmissibility, disease prevalence, extrinsic mortality and rate of care on the prevalence of hominins who prioritized innate immunity when care intensity was set to 0

Predictor	Beta	*T*	*P* value
Transmissibility	0.05	0.11	0.91
Disease mortality	1.15	−1.76	0.09
Extrinsic mortality	0.03	0.83	0.41
Disease prevalence	0.59	1.07	0.29
Care intensity	0.30	−0.87	0.39

#### Care intensity 10

A multiple regression was used to assess the impact of disease mortality, transmissibility, extrinsic mortality, disease prevalence and rate of care on acquired immunity (*M* = 65.7%, SD = 8.62%) when care intensity was set to 10. The regression showed that the predictors accounted for 98% of the variance *F*(5, 38) = 504.2, *P* = 2.2 × 10^−16^ (see Table 6). As disease mortality increased, so did the prevalence of acquired immunity. Hominins who used acquired immunity to combat disease gained immunity for 5 years. In conditions where disease mortality was high, the immunity gained from the use of acquired immunity would have been advantageous to hominins allowing them to collect resources, give care and reproduce with a lower cost to overcoming the disease.

**Table 6.** The effects of disease mortality, transmissibility, disease prevalence, extrinsic mortality and rate of care on the prevalence of hominins who prioritized acquired immunity when care intensity was set to 10

Predictor	Beta	*T*	*P* value
Transmissibility	0.008	0.36	0.72
Disease mortality	0.07	12.8	**4.64 × 10** ^**−16**^
Extrinsic mortality	0.06	−1.29	0.2
Disease prevalence	0.33	−12.4	**1.11 × 10** ^**−1**^
Rate of care	0.21	6.02	**3.68 × 10** ^**−7**^

Bold text highlights significant *P*-value (*P* <0.05).

Transmissibility and extrinsic mortality did not impact the prevalence of acquired immunity. As discussed earlier, the effects of the transmissibility parameter were probably swamped by the effect of re-seeding the disease into the population at a prevalence of 50%. The extrinsic mortality procedure did not activate until the number of hominins in the model was greater than 150. Therefore, the selective pressures of extrinsic mortality were somewhat limited. It is reasonable to suggest that if extrinsic mortality did not have this limitation, it may have impacted the prevalence of acquired immunity.

As the prevalence of acquired immunity increased, the disease prevalence decreased. The employment of acquired immunity provided hominins with 5-time steps of immunity from disease. Therefore, as more individuals used acquired immunity, they gained immunity and overall disease prevalence reduced. Furthermore, as the prevalence of acquired immunity increased, the rate of care increased. As more individuals gained immunity from disease, their resources were not drained from overcoming infection, allowing them to provide care more frequently.

As hominins prioritized either acquired or innate immunity, the impact of the predictors on innate immunity (*M* = 34.3%, SD = 8.62%) mirrored that of acquired immunity *F*(5, 38) = 504.2, *P* = 2.2 × 10^−16^ (see Table 7). Thus, in the model, the effects of the predictors on innate immunity were the opposite of those on acquired immunity.

**Table 7.** The effects of disease mortality, transmissibility, disease prevalence, extrinsic mortality and rate of care on the prevalence of hominins who prioritized innate immunity when care intensity was set to 10

Predictor	Beta	*T*	*P* value
Transmissibility	0.008	−0.36	0.72
Disease mortality	0.07	−12.8	**4.64 × 10** ^**−16**^
Extrinsic mortality	0.06	1.29	0.2
Disease prevalence	0.33	12.4	**1.11 × 10** ^**−1**^
Rate of care	0.21	−6.02	**3.68 × 10** ^**−7**^

Bold text highlights significant *P*-value (*P* <0.05).

#### Care intensity 20

A multiple regression was used to assess the impact of disease mortality, transmissibility, extrinsic mortality, disease prevalence and rate of care on acquired immunity (*M* = 65.2%, SD = 8.29%) when care intensity was set to 20. The regression showed that the predictors accounted for 99% of the variance *F*(5, 42) = 1126, *P* = 2.2 × 10^−16^ (see Table 8). The impact of care intensity being set to 20 was very similar to that of care intensity at 10.

**Table 8.** The effects of disease mortality, transmissibility, disease prevalence, extrinsic mortality and rate of care on the prevalence of hominins who prioritized acquired immunity when care intensity was set to 20

Predictor	Beta	*T*	*P* value
Transmissibility	0.005	−0.20	0.98
Disease mortality	0.04	13.1	**2 × 10** ^**−16**^
Extrinsic mortality	0.05	1.2	0.24
Disease prevalence	0.43	11.6	**2 × 10** ^**−16**^
Rate of care	0.02	−22.8	**1.12 × 10** ^**−14**^

Bold text highlights significant *P*-value (*P* <0.05).

As hominins prioritized either acquired or innate immunity, the impact of the predictors on innate immunity (*M* = 34.8%, SD = 8.29%) mirrors that of acquired immunity *F*(5, 42) = 1126, *P* = 2.2 × 10^−16^ (see Table 9). Thus, in the model, the effects of the predictors on innate immunity were the opposite of those on acquired immunity (see Fig. 2).

**Table 9.** The effects of disease mortality, transmissibility, disease prevalence, extrinsic mortality and rate of care on the prevalence of hominins who prioritized innate immunity when care intensity was set to 20

Predictor	Beta	*T*	*P* value
Transmissibility	0.005	0.20	0.98
Disease mortality	0.04	−13.1	**2 × 10** ^**−16**^
Extrinsic mortality	0.05	−1.2	0.24
Disease prevalence	0.43	−11.6	**2 × 10** ^**−16**^
Rate of care	0.02	22.8	**1.12 × 10** ^**−14**^

Bold text highlights significant *P*-value (*P* <0.05).

### Selection on pace of life

A multiple regression was used to assess the impact of disease mortality, transmissibility, disease prevalence, extrinsic mortality, rate of care and care intensity on the percentage of immature hominins. The regression showed that the predictors accounted for 70% of the variance *F*(6,133) = 53.7, *P* = 2.2 × 10^−16^ (see Table 10). Higher disease prevalence was associated with a lower percentage of the population being immature. Immature hominins collected resources at a lower rate so they have more difficulty acquiring enough resources to overcome diseases and avoid starvation. While care does not have to go from parent to offspring, mature individuals collect more resources and so are more likely to have sufficient resources to provide care. Both rates of care and care intensity have the effect of increasing the survival of the young. As the rate of care increases, more offspring receive care and gain both protection from extrinsic mortality and potentially additional resources. Additional resources when receiving care, enable them not only to overcome disease but also to avoid starvation. Immature hominins are, therefore, disproportionately protected from extrinsic mortality via receiving care. As a result, extrinsic mortality becomes more likely to remove more mature than immature individuals from the population. The percentage of the population that is immature accordingly increases.

**Table 10.** The effects of disease mortality, transmissibility, disease prevalence, extrinsic mortality, rate of care and care intensity on percentage of immature hominins

Predictor	Beta	*T*	*P* value
Transmissibility	0.04	−0.26	0.79
Disease mortality	0.27	0.6	0.55
Extrinsic mortality	0.2	10.7	**2 × 10** ^**−16**^
Disease prevalence	0.08	−6.44	**1.99 × 10** ^**−9**^
Rate of care	0.5	6.09	**1.16 × 10** ^**−8**^
Care intensity	0.18	3.91	**1.48 × 10** ^**−4**^

Bold text highlights significant *P*-value (*P* <0.05).

As hominins were either immature (under 15 years old) or mature (15 years and older), the impact of the predictors on the percentage of mature hominins mirrored that of the percentage of immature hominins (see Table 11).

**Table 11.** The effects of disease mortality, transmissibility, disease prevalence, extrinsic mortality, rate of care and care intensity on percentage of mature hominins

Predictor	Beta	*T*	*P* value
Transmissibility	0.04	0.26	0.79
Disease mortality	0.27	**−**0.6	0.55
Extrinsic mortality	0.2	**−**10.7	**2 × 10** ^**−16**^
Disease prevalence	0.08	6.44	**1.99 × 10** ^**−9**^
Rate of care	0.5	**−**6.09	**1.16 × 10** ^**−8**^
Care intensity	0.18	**−**3.91	**1.48 × 10** ^**−4**^

Bold text highlights significant *P*-value (*P* <0.05).

A multiple regression was used to assess the impact of disease mortality, transmissibility, disease prevalence, extrinsic mortality, rate of care and care intensity on reproductive rate. The regression showed that the predictors accounted for 93% of the variance *F*(6,133) = 304, *P* = 2.2 × 10^−16^ (see Table 12). In conditions where disease prevalence was high, the rate of reproduction decreased. This is likely because hominins were then using their resources to overcome disease or provide care, rather than reproduce. In the model, hominins first combat disease, then give care, then reproduce according to how many remaining resources they have. This represents a prioritization of the parent’s health and raising of existing offspring to maturity over having additional young. Interestingly, the rate of care and care intensity did not significantly influence the reproductive rate. This may indicate that the expense of overcoming disease was the primary determinant of whether hominins had sufficient resources to reproduce.

**Table 12.** The effects of disease mortality, transmissibility, disease prevalence, extrinsic mortality, rate of care and care intensity on reproductive rate

Predictor	Beta	*T*	*P* value
Transmissibility	0.03	1.14	0.25
Disease mortality	0.18	4.09	**7.53 × 10** ^**−5**^
Extrinsic mortality	0.13	11.11	**2 × 10** ^**−16**^
Disease prevalence	0.05	**−**16.51	**2 × 10** ^**−16**^
Rate of care	0.33	2.33	0.21
Care intensity	0.12	**−**1.23	0.22

Bold text highlights significant *P*-value (*P* <0.05).

Increases in both disease mortality and extrinsic mortality have the effect of reducing the population size below carrying capacity. This allows the surviving mature individuals to increase their rate of reproduction (see Fig. 3).

## DISCUSSION

### Overview

Our study found that disease characteristics and levels of extrinsic mortality did interact with care giving behaviors to produce shifts in the immune strategy and pace of life of the hominin population. Our hypothesis that care giving would select for acquired immunity was supported. However, our hypothesis that care giving outcomes of reduced extrinsic mortality, increased pathogen exposure and increased availability of resources to sick/injured individuals would promote selection for a slower pace of life was not supported. The population shifted to a faster, but also cost-intensive, pace of life instead. Below we discuss the complex dynamics that produced these effects and relate the findings to what we know about human evolution. As the dynamics are occurring over time and are likely to produce feedback loops, our interpretations focus more on associations between variables rather than unidirectional statements of causality.

### Selection for acquired immunity

Higher rates of care were associated with higher prevalences of acquired immunity in the population when care included provisioning (resource transfer of 10 or 20). Care, absent of resource transfer (care intensity/resource transfer 0) was not associated with a change in immune strategy. Care intensity 0 included protection from extrinsic mortality but no resource transfer. This, therefore, indicated that protection from extrinsic mortality alone was not sufficient to select for acquired immunity and that the key influencing factor was the transfer of resources through provisioning. This was key to enabling recipients to develop and deploy acquired immunity, which, in turn, greatly reduced the cost of staying healthy for five-time steps, allowing the recipient to collect resources, give care, and/or reproduce without draining their resources to overcome disease. As care was given to kin, and immune strategy was heritable, the acquired immunity strategy increased in the population both through direct reproduction (hominins who used acquired immunity having sufficient resources to reproduce) and through kin selection (transferring resources to kin who were likely also to use acquired immunity and may then reproduce themselves) [24, 69, 70].

In addition, the interplay of care and acquired immunity may have had the effect of controlling the prevalence of disease. Care intensity and rate of care were positively associated with disease prevalence in Table 3, suggesting that the rate and intensity of care were higher when disease prevalence was higher. We did not associate care giving with heightened disease transmission risk in the model. We, therefore, expect that the positive association rate of care with disease prevalence indicated increased care giving in circumstances of increased disease prevalence rather than increasing disease prevalence in circumstances of increased care giving. Significantly, the positive association of care intensity and rate with disease prevalence changed when acquired immunity was incorporated. Higher care intensities (10 and 20) and a higher rate of care were associated with a higher prevalence of acquired immunity, but a **lower** prevalence of disease (Tables 6 and 8). This suggests that when care is given, the hominins that can deploy acquired immunity do so, and then do not transmit the disease for the next five time-steps thereby reducing the prevalence. Thus, by subsidizing the investment of others in their acquired immunity, care giving may reduce disease prevalence in the population.

### Complex effects on the pace of life—shifting to a rapid, high-investment reproductive strategy

The two measures of the pace of life that we used (reproductive rate and percentage of the population that is immature) suggest that in circumstances of increased care giving the population did **not** shift to a slower pace of life as hypothesized. Instead, it shifted to a faster but **more** cost-intensive reproductive strategy. This does not fit neatly into the slow versus fast pace of life spectrum [16–18, 36]. Interestingly, this is similar to shifts that have been argued to have been made in the hominin lineage in the genus *Homo* [16–18].

The rate of reproduction increased suggesting the population shifted toward more rapid reproduction (a characteristic of a faster pace of life) in response to the selective pressures exerted in the model. Reproductive rate increased as extrinsic and disease mortality increased. In ecological terms, greater mortality rates reduced the population size relative to the carrying capacity, enabling healthy hominins with sufficient resources to reproduce. Disease prevalence was negatively associated with reproductive rate, probably because hominins used their resources to overcome disease and were less likely to have enough remaining resources to reproduce. A higher reproductive rate generally represents higher energetic investments, unless the parent is investing less in each individual offspring—but this does not appear to have been the case.

The percentage of the immature hominins on the population increased. High-intensity care giving appears to have increased the survival of the immature hominins, resulting in a higher percentage of the population being immature. Both care intensity and rate of care were positively associated with the percentage of the population that was immature. This is likely to have occurred because care would have protected the recipient from extrinsic mortality, and (during care intensity 10 and 20) from disease mortality and starvation via provisioning with enough resources necessary to mount an immune response and survive. While care was not exclusively given from mature hominins to immature offspring, mature hominins collected more resources per time step so were more likely to have sufficient surplus to be able to provide care. Similarly, as said above, if immature hominins are more likely to be protected from extrinsic mortality, extrinsic mortality is likely to disproportionately reduce the number of mature individuals in the population.

### Relating the model to human evolution

The model’s findings that care led to a shift in reproductive strategy align with interpretations of the early *Homo* fossil record. A change in population dynamics occurs in the fossil record around the emergence of early *Homo* [73]. Previously, hominin groups had a greater proportion of adults than children. However, the prevalence of child fossils dated to this period is much greater than that of adult fossils giving rise to the suggestion that reproduction rates and child mortality were high [73]. When considering this, together with the model’s finding that care can increase child survival, the observed shift in population dynamics may suggest that hominins were facing strong selective pressure to evolve care to reduce child mortality.

For much of human history child mortality has been high with disease being a major cause of death for young children [74]. Others probably included starvation, interpersonal violence, injury, and so on [73]. However, the findings of the model support the idea that early hominin populations, even those potentially providing care, may have had larger numbers of young and those young probably faced high mortality risks. Consequently, hominins may have evolved to invest in frequent, costly care, thus subsidizing the immune development of their young. This reproductive strategy has continued through to modern times with parents who survived various childhood diseases then providing care for their own offspring as they experience them (i.e. measles, small pox, chicken pox, and so on).

This care is likely to have created significant costs for carers. However, evidence from modern small-scale societies suggests that, if ancestral hominins operated in a similar manner to them, they may have overcome the dilemma of the high cost of care through a unique and extensive system of cooperatively provisioning kin and other group members [61]. Hunter-gatherers primarily hunt for large, mobile game and gather other micronutrients [61]. This difficult foraging niche and the provisioning of offspring well into teenage years made food shortages likely [61]. However, food sharing and the division of labor between men and women significantly reduces the risk of individuals going without food [61, 75]. This system of provisioning is argued to have enabled females to increase their rate of reproduction, relative to other primates, which do not cooperatively provision young [16–18].

Moreover, this system of cooperatively provisioning young that evolved in our lineage was likely extended to the provision of care to infected individuals. Evidence from modern small-scale societies suggests that this type of extended, costly provisioning is crucial for enabling sick and injured individuals to survive [64]). For example, Sugiyama [64]estimates that illness and injury are frequent, with most living individuals having experienced health crises that would have been fatal without provisioning. A key aspect of this system is that the cost to any single provider could be reduced by increasing the number of providers, while the benefit to the recipient remained the same.

While our model did not explicitly model cooperative care, it may have produced some emergent effects which suggest that cooperative care would be prone to evolving in care giving populations. While mature hominins collected resources at a faster rate, and were, therefore, more likely to have the surplus needed to provide care, care giving was not restricted to mature hominins. In addition, hominins could only provide care to one individual per time-step, meaning that at times of high disease prevalence, there could be multiple offspring waiting for care. If the parent becomes infected, and some of the offspring have sufficient resources, they could provide care to the parent. This would enable the parent to overcome the disease and, if they use acquired immunity, gain immunity for five time-steps. This parent would then be able to rapidly collect the resources to care for the remaining offspring, who are siblings to the offspring that provided care to the parent. In this way, individuals are able to increase the likelihood of their siblings receiving care. This represents the emergent effect of cooperative care evolving through kin selection. We expect that similar dynamics could have occurred during human evolution.

In conclusion, our findings support the predictions made by McDade *et al.* [35], in that care, particularly provisioning, was selected for greater investment in acquired immune responses. Where our findings differed from McDade’s predictions are that we found that instead of slowing the pace of life, care was associated with a shift toward a rapid, high-investment reproductive strategy. This unexpected effect aligns well with interpretations of the fossil record and ethnographic data of modern small-scale societies suggesting that humans evolved to breed cooperatively. Moreover, our study adds to a growing a body of work demonstrating the value of using agent-based models to refine evolutionary theory about care during human evolution, complement other bodies of evidence (e.g. fossil records, ethnography), and simulate controlled comparisons that would otherwise not be possible [24, 55].

Together, these different streams of evidence suggest that care giving, particularly provisioning, may have selected our species to invest in acquired relative to innate immune responses, with an extended period of immune development being subsidized by care-givers. While all individuals naturally engage in complex immune responses encompassing both acquired and innate responses, immune strategies and trade-offs can be visible in cross-species comparisons [76]. This points to new questions about whether our species may have engaged in immune strategy trade-offs and if we could somehow be immunologically specialized for care giving. These questions could be addressed empirically with future research comparing the processes of immune development across species, particularly other apes.

## Supplementary Material

eoae004_suppl_Supplementary_Appendix_A

eoae004_suppl_Supplementary_Appendix_B
