# Supplementary material for: Could care giving have altered the evolution of human immune strategies?
Source: Evol Med Public Health. 2024 Jan 25;12(1):33–49. doi: 10.1093/emph/eoae004 (PMC10878251; doi:10.1093/emph/eoae004)
Supplement: eoae004_suppl_Supplementary_Appendix_A [file eoae004_suppl_supplementary_appendix_a.docx]

# Appendix A

globals

[

seed ; random seed

total-helped ;total number of times hominins provided care

total-reproduced ; total number of times hominins reproduced

died-of-em ;total number of times hominins died of extrinsic mortality

died-of-starvation ;total number of times hominins died of starvation

died-of-disease ;total number of times hominins died of disease

died-of-sickness ;total number of times hominins died of sickness - sickness defined as running out of resources while combatting disease

remember-disease-timer ;timer for hominins using acquired immunity to run out

used-innate-immunity ;the total number of times hominins used innate immunity

used-acquired-immunity ;the total number of times hominins used acquired immunity

disease-reintroduction ;total number of times the disease was reintroduced

numberturtles_file ;files for recording outcome variable

percentstrat0_file

percentstrat1_file

countinfectectturtles_file

percentyoungagents_file

percentmatureagents_file

countyoungagents_file

countmatureagents_file

diseaseprevalence_file

totalhelped_file

totalreproduced_file

percenthelped_file

percentreproduced_file

]

turtles-own

[

age ;the hominins age variable

investment-strategy ;investment strategy variable

resource-score ;the hominins resource score variable

infected? ;the hominins infection status. If it is set to TRUE, the hominin is infected. If it is set to FALSE, the hominin is not infected

remember-disease? ;the hominins immunity status. If set to TRUE, the hominin is immune. If it is set to FALSE, the hominin does not have immunity

received-care? ;the hominins care status. If set to TRUE, the hominin have received care. If it is set to FALSE, the hominin has not received care

received-protection? ;the hominins protection status. If set to TRUE, the hominin is immune. If it is set to FALSE, the hominin does not have immunity

number-of-offspring ;the number of offspring the hominin has.

]

patches-own

[

resources ;the patches resources

]

;-----------------------------------------------------------------------------------

to set-up

ca

set seed new-seed ;resets the random seed

random-seed seed

reset-ticks ;resets time steps

set died-of-em 0 ;resets recording variables

set died-of-starvation 0 ;resets recording variables

ask patches

[

set resources random 10 ;Randomly sets the patch resources between 0-10

if resources = 0 or resources < 0 or resources < 5[set pcolor red] ;displays the resources visually

if resources > 5 or resources = 5 [set pcolor blue]

if resources = 10 [set pcolor green]]

crt 100

[

set size 1

set shape "circle"

set color white

setxy random-xcor random-ycor ;places the hominins randomly in the model

set resource-score 20 ;sets the hominins resources randomly between 0-20

set age random 60 ;sets age randomly between 0-60

set infected? FALSE ;Setting infection status to not infected

set remember-disease? FALSE ;setting immunity to not immune

set investment-strategy random 2 ;setting immune strategy to 0 or 1 0=prioritising innate immunity, 1= prioritising acquired immunity

set received-care? FALSE ;stating that the agent has not received care

set received-protection? FALSE ;stating that the agent has not received protection

set number-of-offspring 0 ; setting the number of offspring to 0

]

tick

end

;--------------------------------Go Procedure--------------------------------------

;sets the procedures for patches and turtles in this order/ time step

to go

ask patches ;setting patches to perform the following procedures

[

display-resources ;visually displaying the resources available on the patch

grow-resources ;to replenish the number of resources available on a patch

]

ask turtles ;setting hominins to perform the following procedures

[

ifelse infected? = true ;setting hominins to visually display infection

[set color yellow]

[set color blue]

set received-care? FALSE ;stating that the agent has not received care

set received-protection? FALSE ;stating that the agent has not received protection

grow-old

forage

begin-disease

transmit-disease

provide-care

do-extrinsic-mortality

combat-disease

reproduce

reintroduce-disease

forget-disease

starve

]

final-calculations ;setting to model to record the variables listed in the procedure

tick ;to move onto the next time step

if count turtles = 0 [stop] ;if the number of hominins reaches 0, the run stops

if ticks > 750 [stop] ;if the time steps reach 750, the run stops

end

;----------------------------Observations-----------------------------------------------

;calculating reporters throughout the model.

to-report disease-prevelance ;the percent of infected hominins within the model

let no-infected-turtles count turtles with [infected? = TRUE]

let percentage-infected no-infected-turtles / count turtles * 100

report percentage-infected

end

to-report percentage-investment-strat-0 ;the percent of hominins using innate immunity

let n-of-strat-0 count turtles with [investment-strategy = 0]

let percent-of-strat-0 n-of-strat-0 / count turtles * 100

report percent-of-strat-0

end

to-report percentage-investment-strat-1 ;the percent of hominins using acquired immunity

let n-of-strat-1 count turtles with [investment-strategy = 1]

let percent-of-strat-1 n-of-strat-1 / count turtles * 100

report percent-of-strat-1

end

to-report percent-of-agents-helped ;the percent of hominins who received care in a time step - rate of care

let number-of-agents count turtles with [received-care? = true]

if number-of-agents = 0 [report 0]

let percent-helped (number-of-agents / count turtles) * 100

report percent-helped

end

to-report percent-of-young-agents ;the percent of immature hominins in the model

let count-turtles count turtles

let young-turtles count turtles with [age < 15]

let percent young-turtles / count-turtles * 100

report percent

end

to-report percent-of-mature-agents ;the percent of mature hominins in the model

let mature-turtles count turtles with [age >= 15]

if mature-turtles = 0 [report 0]

let percent mature-turtles / count turtles * 100

report percent

end

to-report percent-of-agents-reproduced ;the percent of mature hominins who had reproduce per time step - rate of reproduction

let mature-agents count turtles with [age >= 15]

let parents count turtles with [number-of-offspring > 0 ]

if parents <= 0 [report 0]

let percent-parents parents / mature-agents * 100

report percent-parents

end

;----------------------------------patch procedures--------------------------------------

to display-resources ;visually displaying resources

if resources = 0 or resources < 0 or resources < 5[set pcolor red]

if resources > 5 or resources = 5 [set pcolor blue]

if resources = 10 [set pcolor green]

end

to grow-resources ;replenishing resources

let num-turtles count turtles

if num-turtles > 150 [stop] ;checking the carrying capacity has not been reached

if resources < 10 [set resources 10] ;resetting resources to 10

end

;------------------------- Normal Agent procedures-----------------------------------------

to starve

if resource-score = 0 [set died-of-starvation died-of-starvation + 1 die] ;if the hominin has no resources, they die and record death

if resource-score < 0 [set died-of-starvation died-of-starvation + 1 die] ;if the hominin has less than 0 resources, they die and record death

end

to grow-old

set age age + 1 ;age increases by 1/ time step

if age > 60 [die] ;when the hominin reaches 60, they die

end

to forage

if resource-score > 50 [stop] ;stop collecting resources past 50

if infected? = TRUE [stop] ;cant collect resources if infected

ifelse age < 15 ;if agent is younger than 15 they follow the first set of foraging criteria

[let available-patch patches in-radius 5 with [resources > 5 or resources = 5] ;checking the if there are available patches in a 5 patch radius

if not any? available-patch [stop]

move-to one-of available-patch ;move to and occupy that patch

set resource-score resource-score + 5 ;hominin increase resources by 5

ask patch-here [

set resources resources - 5]] ;patch reduces resources by 5

[let available-patch patches in-radius 5 with [resources = 10] ;foraging procedure for adults, checking the if there are available patches in a 5 patch radius

if not any? available-patch [stop]

move-to one-of available-patch ;move to and occupy that patch

set resource-score resource-score + 10 ;hominin increase resources by 5

ask patch-here [

set resources resources - 10]] ;patch reduces resources by 5

end

;--------------------------------disease procedures---------------------------------------

to begin-disease

if ticks = 5 ;checking that time-steps have reached 5

[

ask n-of (count turtles / 100 * 50) turtles

[ set infected? true ] ;asking 50% of hominins to set infected status to infected

]

end

to transmit-disease

if any? turtles with [infected? = TRUE and remember-disease? = FALSE] ;creating a subset of infected hominins who dont have immunity

[ask turtles with [infected? = TRUE and remember-disease? = FALSE]

[let center self

let centers-risky-patches patches in-radius 5 ;identify the infection radius

ask centers-risky-patches

[if any? turtles-here with [infected? = false] ;identify subset of hominins who are not infected

[let centers-vulnerable-turtles turtles-here with [infected? = FALSE and remember-disease? = FALSE]

ask centers-vulnerable-turtles

[let rand-infect random-float 100 ;select a random number

if rand-infect < transmissibility ;if the number is less than transmissibility hominin sets infected to true and changes colour

[set infected? true

set color yellow ]]]]]]

end

;---------------------------------care-giving----------------------------------------

to provide-care

if not any? link-neighbors with [infected? = TRUE] [STOP] ;checking for infected relatives

let infected-relatives link-neighbors with [infected? = TRUE]

let infected-relative one-of link-neighbors with [infected? = TRUE] ;select one infected relative

set resource-score resource-score - care-factor ;reduce resource score by care intensity

ask infected-relative [

set received-care? TRUE

set received-protection? TRUE

set total-helped total-helped + 1

set resource-score resource-score + care-factor] ;infected relative increases resource score by care intensity

end

to do-extrinsic-mortality

if count turtles >= 150 and received-protection? = FALSE ;checking carrying capacity and if agent is immune to extrinsic mortality

[let rand-em-death random-float 100 ;select random number between 0-100

if rand-em-death < extrinsic-mortality ;if number is less than preset extrinsic mortaltiy

[set died-of-em died-of-em + 1 die]] ;die and record death

end

;------------------------------------------immunity and investment strategy---------------------------------

to combat-disease

if infected? = TRUE ;checking if the hominin is infected

[if investment-strategy = 0 ;if hominin prioritises innate immunity

[let percentage-investment random-float 100 ;generate random number between 0-100

ifelse percentage-investment < 75 [innate-combat-disease] ;if random number is less than 75 use innate immunity

[if percentage-investment > 75 [acquired-combat-disease]]] ;if number is more than 75 use acquired

if investment-strategy = 1 ;if hominin prioritises acquired immunity

[let percentage-investment random-float 100 ;generate random numberbetween 0-100

ifelse percentage-investment < 75 [acquired-combat-disease] ;if number is less than 75 use acquired immunity

[if percentage-investment > 75 [innate-combat-disease]]]] ;if the number is greater than 75 use acquired immunity

end

to innate-combat-disease

let random-death random-float 100 ;generate random numberbetween 0-100

if random-death < disease-mortality ;if number is less than previously sat disease mortality

[set died-of-disease died-of-disease + 1 ;die and record death

die]

ifelse resource-score <= 5 ;check hominin has enough resources to combat infection

[stop] ;if not, stop and move on to next procedure

[set resource-score resource-score - 5 ;reduce resources by 5

if resource-score <= 0 [set died-of-starvation died-of-starvation + 1 die] ;if resources decrease to 0 or less die and record death

set infected? FALSE ;set infection status to not infected

set used-innate-immunity used-innate-immunity + 1] ;record use of innate immunity

end

to acquired-combat-disease

ifelse remember-disease? = TRUE ;checking if hominin has immunity

[set infected? FALSE ;set infection status to not infected

set resource-score resource-score - 1 ;loose one resource point

if resource-score < 0 or resource-score = 0 [set died-of-sickness died-of-sickness die]] ;if hominin runs out of resources, die and record death

[let random-death random-float 100 ;generate random numberbetween 0-100

if random-death < disease-mortality ;if number is less than previously sat disease mortality

[set died-of-disease died-of-disease + 1 die] ;die and record death

ifelse resource-score <= 10 [stop] ;check hominin has enough resources to combat infection

[set resource-score resource-score - 10 ;reduce resources by 10

if resource-score < 0 or resource-score = 0 [set died-of-sickness died-of-sickness + 1 die] ;if resources decrease to 0 or less die and record death

set infected? FALSE ;set infection status to not infected

set remember-disease? TRUE ;set immunity status to immune

set used-acquired-immunity used-acquired-immunity + 1]] ;record use of acquired immunity

end

;-------------------------Normal Agent procedures-----------------------------------------

to reproduce

if age < 15 [stop] ;check age

if age > 45 [stop]

if resource-score < 20 [stop] ;check they have enough resources

if count turtles > 200 [stop] ;check carrying capacity

if infected? = TRUE [stop] ;check infection status

set resource-score resource-score - 20 ;decrease resources by 20

set total-reproduced total-reproduced + 1 ;record reproduction

set number-of-offspring number-of-offspring + 1 ;record offspring

let parent self ;reproduce and set variables

hatch 1

[let chance-of-inheritance random-float 100 ;generate randome number between 0-100

ifelse chance-of-inheritance < 75 ;if number is less than 75

[set investment-strategy [investment-strategy] of parent] ;inherit investment strategy of parent

[set investment-strategy random 2] ;if number is more than 75, randomly assign investment strategy

set color [color] of parent

set size 0.5

set shape "circle"

set age 0

create-link-with parent ;linked to parent for care provision

set resource-score 5

set infected? FALSE

set remember-disease? FALSE

set number-of-offspring 0

]

end

to reintroduce-disease

let number-of-infected-turtles count turtles with [ infected? = TRUE]

ifelse number-of-infected-turtles = 0 ;checking number of infected agents, if 0

[set disease-reintroduction disease-reintroduction + 1 ;record disease reintroduction

ask n-of (count turtles / 100 * 50) turtles ;randomly infected 50% of population

[set infected? TRUE

set remember-disease? FALSE]]

[stop]

end

;------------------------------------------immunity and investment strategy---------------------------------

to forget-disease

set remember-disease-timer remember-disease-timer - 1 ;reduce disease timer by 1

if remember-disease-timer = 0 ;when it reaches 0

[

set remember-disease? FALSE ;loose immunity

reset-forget-disease ;reset disease timer

]

end

to reset-forget-disease

ifelse remember-disease-timer = 0 ;when timer reaches 0

[set remember-disease-timer 5 ] [stop] ;reset to 5

end

to final-calculations ;collecting the final calculations

let count-turtles count turtles

let count-infected-turtles count turtles with [infected? = TRUE]

let count-young-turtles count turtles with [age < 15]

let count-mature-turtles count turtles with [age >= 15]

if ticks = 1 ;creating the files to record variables per time step

[ ;setting output file names.

set numberturtles_file (word "numberofturtles" " " seed " " behaviorspace-experiment-name behaviorspace-run-number " " disease-mortality "-" transmissibility "-" extrinsic-mortality "-" care-factor ".txt")

set percentstrat0_file (word "percentstratinnate" " " seed " " behaviorspace-experiment-name behaviorspace-run-number " " disease-mortality "-" transmissibility "-" extrinsic-mortality "-" care-factor ".txt")

set percentstrat1_file (word "percentstratacquired" " " seed " " behaviorspace-experiment-name behaviorspace-run-number " " disease-mortality "-" transmissibility "-" extrinsic-mortality "-" care-factor ".txt")

set countinfectectturtles_file (word "countinfectectturtles" " " seed " " behaviorspace-experiment-name behaviorspace-run-number " " disease-mortality "-" transmissibility "-" extrinsic-mortality "-" care-factor ".txt")

set percentyoungagents_file (word "percentyoungagents" " " seed " " behaviorspace-experiment-name behaviorspace-run-number " " disease-mortality "-" transmissibility "-" extrinsic-mortality "-" care-factor ".txt")

set percentmatureagents_file (word "percentmatureagents" " " seed " " behaviorspace-experiment-name behaviorspace-run-number " " disease-mortality "-" transmissibility "-" extrinsic-mortality "-" care-factor ".txt")

set countyoungagents_file (word "countyoungagents" " " seed " " behaviorspace-experiment-name behaviorspace-run-number " " disease-mortality "-" transmissibility "-" extrinsic-mortality "-" care-factor ".txt")

set countmatureagents_file (word "countmatureagents" " " seed " " behaviorspace-experiment-name behaviorspace-run-number " " disease-mortality "-" transmissibility "-" extrinsic-mortality "-" care-factor ".txt")

set diseaseprevalence_file (word "diseaseprevalence"" " seed " " behaviorspace-experiment-name behaviorspace-run-number " " disease-mortality "-" transmissibility "-" extrinsic-mortality "-" care-factor ".txt")

set totalhelped_file (word "totalhelped"" " seed " " behaviorspace-experiment-name behaviorspace-run-number " " disease-mortality "-" transmissibility "-" extrinsic-mortality "-" care-factor ".txt")

set totalreproduced_file (word "totalreproduced"" " seed " " behaviorspace-experiment-name behaviorspace-run-number " " disease-mortality "-" transmissibility "-" extrinsic-mortality "-" care-factor ".txt")

set percentreproduced_file (word "percentreproduced"" " seed " " behaviorspace-experiment-name behaviorspace-run-number " " disease-mortality "-" transmissibility "-" extrinsic-mortality "-" care-factor ".txt")

set percenthelped_file (word "percenthelped"" " seed " " behaviorspace-experiment-name behaviorspace-run-number " " disease-mortality "-" transmissibility "-" extrinsic-mortality "-" care-factor ".txt")

]

file-open numberturtles_file

ifelse (count-turtles >= 0) ;checking there is a variable to report for this time step (same throughout variables)

[file-write count-turtles ] ;writing in the file (same throughout variables)

[file-write " "]

if count turtles <= 0 [file-print "0"]

if ticks = 750 [file-print " "] ;stop recording at 750 time steps (same throughout variables)

file-close

file-open percentstrat0_file

ifelse (percentage-investment-strat-0 >= 0)

[file-write percentage-investment-strat-0 ]

[file-write "0"]

if ticks = 750 [file-print " "]

file-close

file-open percentstrat1_file

ifelse (percentage-investment-strat-1 >= 0)

[file-write percentage-investment-strat-1 ]

[file-write "0"]

if ticks = 750 [file-print " "]

file-close

file-open countinfectectturtles_file

ifelse (count-infected-turtles >= 0)

[file-write count-infected-turtles ]

[file-write "0"]

if ticks = 750 [file-print " "]

file-close

file-open percentyoungagents_file

ifelse (percent-of-young-agents >= 0)

[file-write percent-of-young-agents ]

[file-write "0"]

if ticks = 750 [file-print " "]

file-close

file-open percentmatureagents_file

ifelse (percent-of-mature-agents >= 0)

[file-write percent-of-mature-agents ]

[file-write "0"]

if ticks = 750 [file-print " "]

file-close

file-open countyoungagents_file

ifelse (count-young-turtles >= 0)

[file-write count-young-turtles ]

[file-write "0"]

if ticks = 750 [file-print " "]

file-close

file-open countmatureagents_file

ifelse (count-mature-turtles >= 0)

[file-write count-mature-turtles ]

[file-write "0"]

if ticks = 750 [file-print " "]

file-close

file-open diseaseprevalence_file

ifelse (disease-prevelance >= 0)

[file-write disease-prevelance ]

[file-write "0"]

if ticks = 750 [file-print " "]

file-close

file-open totalhelped_file

ifelse (total-helped >= 0)

[file-write total-helped ]

[file-write "0"]

if ticks = 750 [file-print " "]

file-close

file-open totalreproduced_file

ifelse (total-reproduced >= 0)

[file-write total-reproduced ]

[file-write "0"]

if ticks = 750 [file-print " "]

file-close

file-open percenthelped_file

ifelse (percent-of-agents-helped >= 0)

[file-write percent-of-agents-helped]

[file-write "0"]

if ticks = 750 [file-print " "]

file-close

file-open percentreproduced_file

ifelse (percent-of-agents-reproduced >= 0)

[file-write percent-of-agents-reproduced]

[file-write "0"]

if ticks = 750 [file-print " "]

file-close

end
