# Supplementary material for: Could care giving have altered the evolution of human immune strategies?
Source: Evol Med Public Health. 2024 Jan 25;12(1):33–49. doi: 10.1093/emph/eoae004 (PMC10878251; doi:10.1093/emph/eoae004)
Supplement: eoae004_suppl_Supplementary_Appendix_B [file eoae004_suppl_supplementary_appendix_b.docx]

# **Appendix B Model description**

**Initialisation**.

The model is first initiated by setting the parameters for each patch and each hominin at the beginning of the model run as follows:

Patches

- Resources: set the resources of the patch to a random number between 0-10
  - If the resources are between 0-5 set the colour of the patch is set to red.
  - If the resources are equal to or greater than 5 set the colour of the patch is set to blue.
  - If the resources are equal to 10 set the colour of the patch to green.

Hominins

- Size: set the size of the hominin to 1
- Shape: set the shape of the hominin to circle
- Location: randomly place the hominins across the environment
- Age: set each hominin an age between 0 and 60 randomly.
- Infection: set each hominin an infection status at 0
- Investment strategy: set the immune investment strategy of each hominin to innate or acquired immunity randomly.
- Care: set the received care variable of each hominin to “false” as no hominins have received care yet.
- Infection: set the infection status variable of each hominin to “false” as no hominins have been infected yet.
- Protection: set the protection variable of each hominin to “false” as no hominins have received protection yet.
- Number of offspring: set the number of offspring of each hominin to 0 as no hominins have reproduced yet.

The hominins perform the following procedures in the following order during each time step:

- **Age** (the procedure by which hominins increase in age): Hominins increased their age variable by 1.
- **Forage** (the procedure by which the hominins collect resources): Hominins collected resources from patches according to their age and the availability of patches.
- **Begin Disease** (the procedure by which a hominin starts the infection): A hominin will count the number of infected individuals in the model. If there are no infected individuals, the hominin will ask 50% of the other hominins to set their infection status to infected. This procedure does not begin until the time-step counter reaches 5-time steps.
- **Transmit Disease** (the procedure by which hominins infect other individuals): The hominin checks if its own infection status is infected and whether it has immunity to the disease. If the hominin is infected and does not have immunity, it will check if there are any other hominins within a 5 patch radius of itself. If there are hominins in the 5 patch radius who are not infected the transmitting hominin selects a random number between 0-100, if this number is less than the preset chance of transmission the other hominins in the 5 patch radius set their infected status to infected.
- **Provide Care** (the procedure by which hominins transfer resources to infected kin and provide protection from extrinsic mortality). The hominin checks if it has any infected kin. If the hominin has multiple kin, it selects one. This receiving relative then sets its protection variable to “true” as it has received protection. The receiving relative also increases its resources by the previously set care intensity. The care provider reduces its resources by the previously set care intensity.
- **Extrinsic Mortality** (the procedure by which the hominin experiences extrinsic mortality). A hominin counts the number of hominins in the model. If it is greater than or equal to 150 and the hominin has not received care, the model randomly generates a number between 0-100. If this number is less than the previously set extrinsic mortality, the hominin dies.
- **Combatting Disease** (the procedure by which hominins attempt to over-come infection). The hominin checks whether it is infected. If it is infected and it prioritises innate immunity, the model then generates a random number between 0-100. If that number is less than 75, the hominin will use innate immunity to combat disease. If the number is greater than 75, the hominin will use acquired immunity to combat disease. If the hominin prioritises acquired immunity, the opposite will occur. The model will generate a random number between 0-100 and if this number is less than 75, the hominin will use acquired immunity. If the number is more than 75, the hominin will use innate immunity.
- **Combatting Infection Using Innate Immunity**. The model generates a random number between 0-100. If this number is less than the previously set disease mortality then the hominin dies. If the number is greater than the previously set disease mortality, the hominin goes on to check if it has enough resources to overcome infection. If it does not have the resources, it remains infected and moves onto the next procedure in its infected condition. If it has more than 5 resources, it reduces its resources by 5 and sets its infection status to “false”. If over-coming its infection reduces its resources to 0, the hominin dies.
- **Combatting Infection Using Acquired Immunity**. The hominin first checks if it has immunity. If it has immunity, it reduces its resources by 1, sets its infection status to false and moves on to the next procedure. If it does not have immunity, the model generates a random number between 0-100. If this number is less than the previously set disease mortality, the hominin dies. If the number is greater than the previously set disease mortality, the hominin goes on to check if it has enough resources to over-come infection. If it does not have enough resources, it remains infected and moves on to the next procedure in its infected condition. If it does have enough resources, it sets its infection status to “false”, removes 10 resources and sets its immunity to “true”. If using these resources reduces its resource score to 0, the hominin dies.
- **Reproduction** (the procedure by which hominins reproduce). The hominin checks that it is older than 15 and younger than 45, if it has enough resources (20), whether it is uninfected and if the model has reached carrying capacity (200 individuals). If none of these criteria are met, the hominin stops and moves onto the next procedure. If the criteria are all met the hominin reproduces asexually. The offspring are identical to their parents in all settings except immune strategy. For immune strategy, the offspring generate a random number between 0-100. If the number is less than 75, the child inherits its parents’ immune strategy, if the number is greater than 75, the immune strategy is randomly assigned. The hominins’ offspring are “linked” to their parent and care is provided via this link.
- **Reintroduction of Disease** (the procedure by which disease was reintroduced if the number of infected individuals reached 0). The hominin counted the number of infected agents in the model. If there were 0 infected agents, the hominin asked 50% of the total number of hominins in the model to set their infected status to “true”.
- **Lose Immunity** (the procedure by which hominins lose their immunity to disease). If the hominin remembers the disease, the hominin removes 1 point from its 5 step immunity timer. As soon as its immunity timer reaches 0, it loses its immunity and the timer resets and waits for the hominin to register that they remember the disease again.
- **Starvation** (the procedure by which hominins die if they do not have adequate resources). The hominin checks if its resources are equal to or less than 0. If its resources are equal to or less than 0, the hominin dies.

The patches performed the following procedures in the following order during each time step:

**Display Resources** (the procedure by which patches display the number of resources they have). If the resources of a patch are between 0-5 the colour of the patch is set to red. If the resources are equal to or greater than 5 set the colour of the patch to blue. If the resources are equal to 10 set the colour of the patch to green.

Grow resources (the procedure by which the patches increase resources). The patch counts the number of hominins in the model. If the number of hominins in the model is greater than 150, the procedure stops. If the number of hominins is less than 150, the patch re-sets its resources to 10.

**The Behaviour Space Tool.**

The behaviour space tool allows the user to pre-set a list of parameters and repeat the parameters a number of times automatically. We programmed the behaviour space too to vary disease mortality from 0 to 15 in intervals of 5, transmissibility from 25 to 75 in intervals of 25, care intensity from 0 to 20 in intervals of 10 and extrinsic mortality was set to 0, 1, 5 and 10. The tool ran each experiment for 750 time steps and the following outcome variables were record per time step in txt files:

- Number of hominins
- Percent of hominins prioritising acquired immunity
- Percent of hominins prioritising innate immunity
- Number of infected turtles
- Percent of immature hominins
- Percent of mature hominins
- Count of young hominins
- Count of mature hominins
- Disease prevalence
- Total number of time hominins provided care
- Total number of times hominins reproduced
- The rate of reproduction
- The rate of care

The tool then repeated each variable combination 10 times. We repeated this process 10 times to create 100 repletion's of the same variable combinations.
